# On transcending the impasse of respiratory motion correction applications in routine clinical imaging - a consideration of a fully automated data driven motion control framework

**DOI:** 10.1186/2197-7364-1-8

**Published:** 2014-06-17

**Authors:** Adam L Kesner, Paul J Schleyer, Florian Büther, Martin A Walter, Klaus P Schäfers, Phillip J Koo

**Affiliations:** Division of Nuclear Medicine, Department of Radiology, Anschutz Medical Campus, University of Colorado Denver, 12700 E 19th Ave, Box C-278, Aurora, CO 80045 USA; Division of Imaging Sciences and Biomedical Engineering, King’s College London, London, WC2R 2LS UK; European Institute for Molecular Imaging, University of Münster, Münster, 48149 Germany; Institute of Nuclear Medicine and Department of Clinical Research, University Hospital Bern, Bern, 3010 Switzerland

**Keywords:** Respiratory gating, Motion correction, Motion control framework, Data-driven gating, Signal optimization, PET

## Abstract

Positron emission tomography (PET) is increasingly used for the detection, characterization, and follow-up of tumors located in the thorax. However, patient respiratory motion presents a unique limitation that hinders the application of high-resolution PET technology for this type of imaging. Efforts to transcend this limitation have been underway for more than a decade, yet PET remains for practical considerations a modality vulnerable to motion-induced image degradation. Respiratory motion control is not employed in routine clinical operations. In this article, we take an opportunity to highlight some of the recent advancements in data-driven motion control strategies and how they may form an underpinning for what we are presenting as a *fully automated data-driven motion control framework*. This framework represents an alternative direction for future endeavors in motion control and can conceptually connect individual focused studies with a strategy for addressing big picture challenges and goals.

**Electronic supplementary material:**

The online version of this article (doi:10.1186/2197-7364-1-8) contains supplementary material, which is available to authorized users.

## Background

### Communication

The issue of patient respiratory motion in nuclear medicine imaging has been recognized as a significant problem [1, 2]. Most nuclear medicine imaging procedure acquisition times span over minutes. When imaging is acquired at regions near the thorax, the images will likely suffer from respiratory motion artifacts, resulting in lower resolution, detectability, and localization capacity, as well as potential attenuation correction artifacts [3-5]. Studies have shown that patients’ diaphragms can move as much as 1 to 6 cm during scan acquisition [6, 7]. This issue has been acknowledged as a major obstacle in the application and advancement of high-resolution imaging technology and is the current resolution-limiting factor in nuclear medicine thorax imaging [8, 9]. The issue of motion must be resolved to fully utilize presently available high-resolution technologies as well as future innovations. Ideally, the solution should be simple to apply and robust [2, 8].

## Case presentation

Over the last decade, a large body of work has been created developing equipment and strategies for respiratory motion control, primarily using gating [2, 10-12] or breath-hold approaches [13, 14]. Integrated respiratory gating equipment and software are widely available today; research and clinical studies have shown the potential benefit of implementing motion control strategies; yet, despite positive research results, respiratory motion control is still rarely used in routine clinical imaging.

For the adoption and acceptance of motion control, strategies would, for practical considerations, require a favorable cost-to-benefit ratio to be established. Unfortunately, this goal is proving elusive. Respiratory motion control has, by nature, many case-specific factors and uncertainties involved in the gating process as well as the proceeding utilization of gated images, and this obfuscates efforts to characterize both the cost and benefit sides of the cost-benefit ratio. At present, the field lacks a unified vision for addressing this. It is with these considerations in mind that we present the idea that data-driven motion control strategies may have the requisite qualities to surmount this hurdle and drive this area of technology forward.

**Figure 1:**
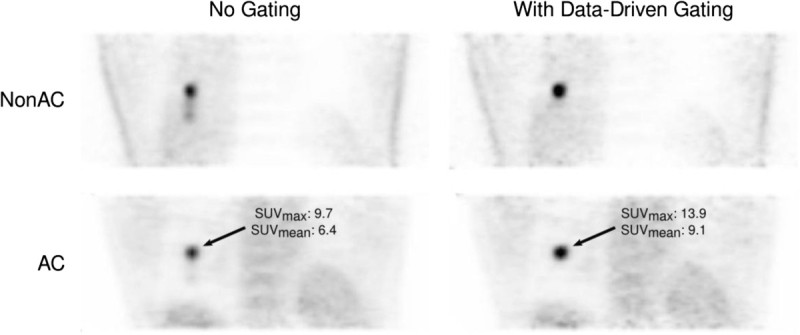
**Example FDG PET images reconstructed without (left) and with (right) data-driven respiratory gating.** Top row images are reconstructed without attenuation correction (AC); bottom row images were corrected for attenuation.

In the last decade, the main focus in this field has been set on establishing and utilizing hardware-based gating strategies. However, in parallel, some less known data-based methods have been developed and refined. These methods utilize untapped information contained in raw acquisition data for image enhancement. Unlike hardware, they can be run without any impact on image acquisition protocols. These strategies have evolved enough with respect to speed and accuracy that data-driven gating appears to have the capacity to perform comparably to hardware [15-18]. An example static positron emission tomography (PET) scan retrospectively gated using data-driven gating methods is shown in Figure 1, illustrating the qualitative and quantitative information this technology can provide with respect to characterizing metabolically active lesions.

Beyond the ability to replace hardware, data-driven algorithms can provide a foundation for an entirely new paradigm of motion control strategies, a paradigm in which we focus on minimal impact and maximal benefit at both ends of the best case scenario (images exhibit obvious benefit) and worst case scenario (effort provides no benefit) spectrum. This is achieved through the integration of both data-driven information capture and information utilization strategies into black box workflows. By expanding the application of motion control beyond highly specific, and potentially self-selective, research cohorts to general, non-specific imaging populations, we could address and ease the concern of whether gating will cause the potential degradation of images and subsequently patient care. To put it simply, the choice of whether to employ motion control could be made easy if the effort and risk of introducing it to clinical operations came close to nil and the potential benefit realization guaranteed. Patients move with different sound ambitious, it is a reasonable trajectory from the sub-field’s current published achievements.

The characterization of a patient’s breathing patterns is presently the only step in the motion control workflow which requires interruption of the traditional clinical workflow, when using vendor-supported hardware. Compared side by side, the implementation of data- and hardware-driven gating has some notable differences; this is illustrated in Table 1. A summary of listed data-driven gating achievements is shown in Table 2 and provides a snapshot of the field’s establishment and progression towards greater speed, accuracy, and practicality.

**Table 1:** **Considerations for implementing different gating strategies**

	Hardware-driven strategies	Data-driven gating
Requires changes to clinical image acquisition procedures	x	
Requires additional hardware	x	
Requires additional software	x	x
Requires additional setup time	x	
Prone to setup error	x	
Information irrecoverable if acquisition error	x	
Decreases clinical throughput	x	
Requires additional training of technologists	x	
Increases radiation exposure to technologists	x	
May cause patient discomfort	x	
Require further establishment before routine clinical use	x	x
Reproducible		**✓**
Operator independent		**✓**
Can be acquired and reacquired from an existing data set (if needed)		**✓**
Non-specific to scan/machine/institution		**✓**
Driven with internal motion		**✓**

**Table 2:** **Summary of publications/accomplishments in fully automated-data driven gating**

Year	Author	Journal/conference	Title	Summary	Attenuation correction	Computer to hardware	Number of patient scans	Studied radiotracers
2001	Klein et al. [19]	IEEE workshop	Fine-scale motion detection using intrinsic list mode PET information	Introduction of axial DD center-of-mass strategy for respiratory motion characterization in cardiac imaging	Yes	Yes	12	FDG
2003	Schleyer et. al. [20]	US patent	Data driven motion correction for nuclear imaging	Introduction of DD masking strategy for respiratory gating in NM imaging	No	No	-	-
2007	Kesner et. al. [21]	SNM annual conference	Respiratory gated PET based on time activity curve analysis	Introduction of DD sinogram voxel fluctuation method	No	No	Sim	FDG
2008	He et. al. [22]	IEEE TNS	A novel method for respiratory motion gated with geometric sensitivity of the scanner in 3D PET	Introduction of DD geometric sensitivity method	Yes	No	1 + sim	FDG
2009	Schleyer et. al. [23]	PMB	Retrospective data-driven respiratory gating for PET/CT	Introduction of "spectral analysis" approach to optimal signal acquisition	Yes	Yes	4	FDG
2009	Kesner et. al. [24]	IEEE TNS	Respiratory gated PET derived in a fully automated manner from raw PET data	Introduction of "image voxel fluctuation" approach to optimal signal acquisition	No	Yes	24	FDG
2009	Büther et al. [25]	JNM	List mode-driven cardiac and respiratory gating in PET	Comparison of multiple methods for hardware- and data-driven gating, also cardiac gating	No	Yes	29	FDG
2010	Büther et. al. [15]	EJNMMI	Detection of respiratory tumor motion using intrinsic list mode-driven gating in positron emission tomography	Extended GSG method, compared multiple methods for gating	Yes	Yes	34	FDG
2010	Kesner et. al. [26]	Medical Physics	A new fast and fully automated software based algorithm for extracting respiratory signal from raw PET data and its comparison to other methods	Introduced ultra-fast processing, compared multiple methods for gating	No	Yes	22	FDG
2011	Schleyer et. al. [16]	PMB	Extension of a data-driven gating technique to 3D, whole body PET studies	Extended *spectral analysis* DD gating method to 3D WB PET	Yes	Yes	11	FDG
2011	Thielemans et. al. [27]	IEEE NSS-MIC	Device-less gating for PET/CT using PCA	Use of PCA to extract respiratory signal from raw PET and CT	No	Yes	6	FDG, FLT
2013	Büther et. al. [28]	EJNMMI	External radioactive markers for PET data-driven respiratory gating in positron emission tomography	Compared multiple methods and reexamined data driven gating utilizing external markers	Yes	Yes	30	FDG
2013	Kesner et. al. [18]	EJNMMI research	Gating, enhanced gating, and beyond: information utilization strategies for motion management, applied to preclinical PET	Extended fast DD motion control methods to preclinical PET, multiple radiotracers, large subject population	No	Yes	84 (rats)	FDG, DMDPA, NH_3_, choline, NaF, FEDPMA, ML10
2013	Schleyer et. al. [29]	IEEE NSS-MIC 2013	Extracting a respiratory signal from raw dynamic PET data that contain tracer kinetics	Extended data-driven gating to dynamic PET/tracer kinetics	No	Yes	53	NH_3_

Table does not include contributions from semi-automated algorithm innovators. DD = data driven.

We can note that most data-driven gating research to date has focused on respiratory and not on cardiac motion. One reason for this is that electrocardiography (ECG) signals, used for gating, work well. ECG gating is an established technology, relatively inexpensive, and reliable. Similar to respiratory motion correction, data driven cardiac gating may offer practical advantages and may be further developed in the future.

Beyond gating, there persists the question on how best to utilize gated data. Respiratory gating provides an uncertain value, particularly in general, non-specific populations. When applying gating, there is a fundamental tradeoff between the potential improved resolution and increased noise resulting from sub-sampled statistics, which affects detectability and contrast-to-noise. It is very difficult to predict who benefits, in what way, and who does not. Patients move in different patterns and to different extents, and available count statistics vary from patient to patient and scanner to scanner.

Different strategies for signal optimization and utilization have been in development in recent years, to limit image degradation caused by the gating processes. Automated data-driven methods can be used to combine separated gates [30], reconstruct data in four dimensions [31], with motion-driven super resolution techniques [9], or strategically filtered so as to only allow statistically supported frequencies to modify baseline ungated images, e.g., automated, on the fly quality assurance [18].

**Figure 2:**
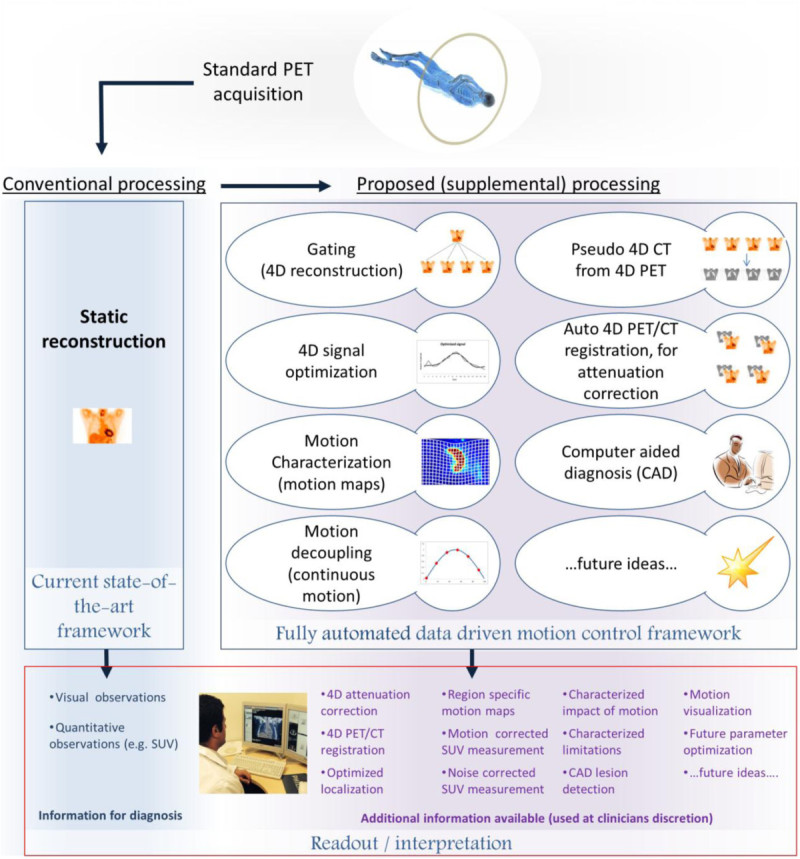
**Flowchart elucidating principle of a fully automated data-driven motion control framework.** Extra information can be provided to the reading physician through additional ‘black box’ processing, integrated with image reconstruction.

**Figure 3:**
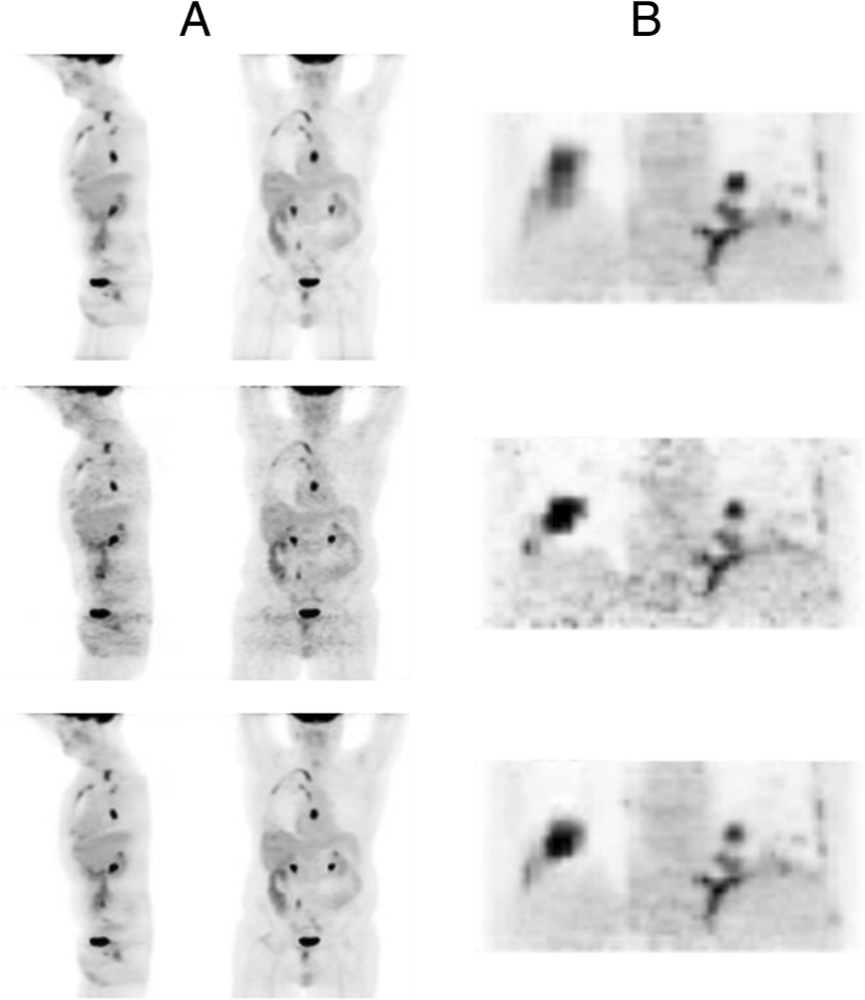
**Example static FDG PET acquisitions processed with data driven gating and signal optimization.** **(A)** Whole body FDG PET scan. **(B)** FDG PET scan attenuation corrected with (PET-) driven gated CT. Top row: vendor reconstruction of non-gated acquisition. Middle row: gated image derived from data-driven gating applied to non-gated acquisition. Bottom row: optimized gated image created through signal optimization procedure applied to the gated images derived from data-driven gating. See Additional files 1 and 2 for cine loop illustrating the motion information captured in the gated and optimized gated images.

Data-driven motion characterization (gating) and signal utilization strategies can readily be combined into application workflows. The concept of building entire image processing workflows with exclusively data-driven components can provide elegant and complimentary solutions for the motion control process and is the basis for defining the data driven motion control framework. This framework could support low-impact solutions for motion control/additional information extraction and is exemplified in Figure 2. For example, the combination of data-driven gating and data-driven quality assurance strategies provides an approach to implement robust motion control through a process of extracting the motion information from raw PET data and using it only if, when, and where it adds value. This process has been shown to work smoothly, and fully automatically, in a population of traditional (non-gated) small-animal PET scans [18]. Presented here are examples, in Figure 3 and Additional files 1 and 2, showing that these workflows could readily be extended to thorax and whole body human imaging. Effectively, the scans or areas of scans that benefit from gating are gated. The scans that do not benefit from gating, because they contain no motion, erratic motion, or low count statistics, can be processed and automatically reverted back to their ungated (i.e., optimal) embodiment. Either way, enhanced images are created with no extra effort put in by patients and technologists and are available for consideration to the reading physicians for use at their discretion.

The future potential of data-driven motion control is promising; we have not seen limits on the information that can be extracted from data nor in the potential innovations that may be made within the automated software framework. We can envision the development of advanced information capture techniques coupled with iterative optimization procedures built to ensure maximum motion control benefit. Processing strategies can be built to support optimal gating/image reconstruction parameter determinations (for example number of gates), motion mapping, image morphing, computer-aided diagnosis (CAD), and continuous-over-time voxel value estimations (Additional files 1 and 2 and [18]). In another exciting direction, gated PET can be used to map static CT scans to PET gates, which in turn can be used to attenuation correct the gated PET, thereby facilitating gated PET and CT without deviating from standard PET-CT protocol (Additional file 2 and Figure 3B). These ideas can also be built to capitalize on the additional information available with new technologies, including time-of-flight PET and PET/magnetic resonance (MR). The resulting implications of ubiquitous data-driven motion control could be improved SUV measurements, localization, lesion detection and delineation, CAD applications, motion characterization, among other uses, - all while imposing no changes to routine imaging procedures.

This article is focused on the potential of combining data-driven technologies. However, it is worth noting also that development within the framework of data-driven motion control can readily extend and be integrated with hardware-based systems. For example, motion mapping, signal optimization, or PET-driven 4D CT modules can be used with hardware-gated scans as easily as they are used with data-driven scans. Furthermore, the developed ability to extract information from raw data may provide hybrid strategies in which data-driven and hardware-driven strategies support and/or back each other up.

The recent emergence of PET/MR technology is also relevant to the discussion of motion control and its future. MR units are capable of providing many kinds of information. Their integration with PET introduces new capacity for potentially robust motion correction. Advancements in this area of technology are already taking place [32, 33]. However, it remains questionable how useful this technology will be for the majority of users in our field, as there are significant costs associated with purchasing, maintaining, and researching PET/MR. At the time of submission, vendor-supplied quotes indicated the price of a PET/MR system to be three times the cost of a TOF PET/CT system.

Data-driven gating and motion control stands in stark contrast to PET/MR with respect to its accessibility. In fact, this area uniquely presents an opportunity for advancing the state-of-the-art technology while decreasing the equipment requirements (and presumably monetary costs). In considering the cost of development, the fully automated data-driven motion control framework is a technology that readily can be developed and implemented within current infrastructures. Research data will come from existing scans and scanners. The entire data-driven sub-field is founded on the fact that there is real information embedded in PET data, information about motion, which is not currently being utilized. The accomplishments in Table 2 attest to this verity.

A recent review article written by Dao well summarizes in its title an opinion that is largely held in our field: ‘Respiratory motion handling is mandatory to accomplish the high-resolution PET destiny’ [8]. Similar sentiments, speaking to the need for practical motion control solutions, have been well articulated in a recent review article by our colleagues in radiation oncology: ‘RG 4D-PET/CT seems to be a valuable tool in improving diagnostic performance of PET/CT and better defining the target volume for radiation therapy. However, its real benefit in routine clinical setting and its possible impact on patient management have not been established yet. In order to bring this technique into the normal workflow of a diagnostic imaging department, simple procedures for scanner setting, fast acquisition protocols and powerful reconstruction-processing algorithms are needed’. [34]. These ideas are shared by others as well [35]. It is clear that there is a desire to develop PET as a dependable 4D modality.

## Conclusions

Respiratory motion has been acknowledged as a problem in nuclear medicine imaging, and there have been calls for the development of effective robust solutions for handling motion control. Data-driven motion control techniques are still only minimally developed, but may offer fast, inexpensive, and potentially robust systems to heed this call. This area can be developed with minimal resources, and one day may extend into the clinic with the assurance of no risk or changes to clinical procedures. To provide structure to this sub-field’s collective efforts and aspirations, we are presenting the concept of a *fully automated data-driven motion control framework*. This framework can provide a conceptual vehicle in which individual concentrated efforts in data-driven processing can readily be integrated into useful, coherent workflows with support a big picture strategy. Furthermore, this framework presents an alternative strategy for addressing the practical problems of respiratory motion in PET, a strategy based on developing low-impact and robust applications and should be appreciable by all stake holders: clinicians, researchers, vendors, and patients aline.

## Ethics statement

All human studies presented in this communication have been approved by the appropriate ethics committees and have been performed in accordance with the ethical standards laid down in the 1964 Declaration of Helsinki and its later amendments. This included approval from the national ethics committee for data acquired at King's College, London, and the ethics committee of the University hospital of Münster, for the data acuired at the University hospital of Münster.

## Electronic supplementary material

Additional file 1: **Whole body FDG PET scan motion animation.** Top row: vendor reconstruction of non-gated acquisition. Middle row: gated image derived from data-driven gating applied to non-gated acquisition. Bottom row: optimized gated imagecreated through signal optimization procedure applied to the gated images derived from data-driven gating. (GIF 5 MB)

Additional file 2: **FDG PET motion animation for PET- driven attenuation corrected data set.** Top row: vendor reconstruction of non-gated acquisition. Middle row: gated image derived from data-driven gating applied to non-gated acquisition. Bottom row: optimized gated image created through signal optimization procedure applied to the gated images derived from data-driven gating. The gated images displayed were corrected for attenuation by generating a pseudo 4D CT attenuation map derived from 4D PET motion fields. (GIF 5 MB)

Below are the links to the authors’ original submitted files for images.

Authors’ original file for figure 1

Authors’ original file for figure 2

Authors’ original file for figure 3

Authors’ original file for figure 4
