# Supplementary figures and images for: On transcending the impasse of respiratory motion correction applications in routine clinical imaging - a consideration of a fully automated data driven motion control framework
Source: EJNMMI Phys. 2014 Jun 17;1:8. doi: 10.1186/2197-7364-1-8 (PMC4673082; doi:10.1186/2197-7364-1-8)

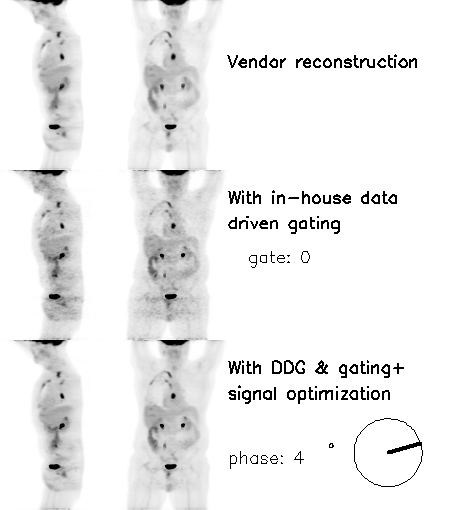

Supplement: Supplementary file 1 — Additional file 1: Whole body FDG PET scan motion animation. Top row: vendor reconstruction of non-gated acquisition. Middle row: gated image derived from data-driven gating applied to non-gated acquisition. Bottom row: optimized gated imagecreated through signal optimization procedure applied to the gated images derived from data-driven gating. (GIF 5 MB) [file 40658_2014_7_MOESM1_ESM.gif]

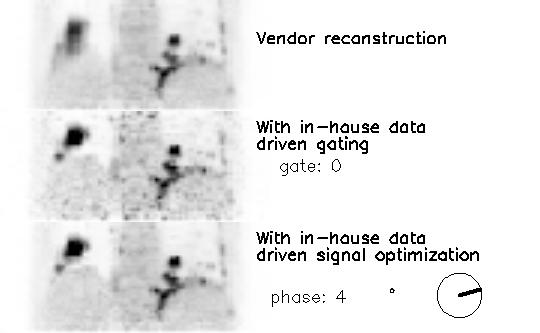

Supplement: Supplementary file 2 — Additional file 2: FDG PET motion animation for PET- driven attenuation corrected data set. Top row: vendor reconstruction of non-gated acquisition. Middle row: gated image derived from data-driven gating applied to non-gated acquisition. Bottom row: optimized gated image created through signal optimization procedure applied to the gated images derived from data-driven gating. The gated images displayed were corrected for attenuation by generating a pseudo 4D CT attenuation map derived from 4D PET motion fields. (GIF 5 MB) [file 40658_2014_7_MOESM2_ESM.gif]

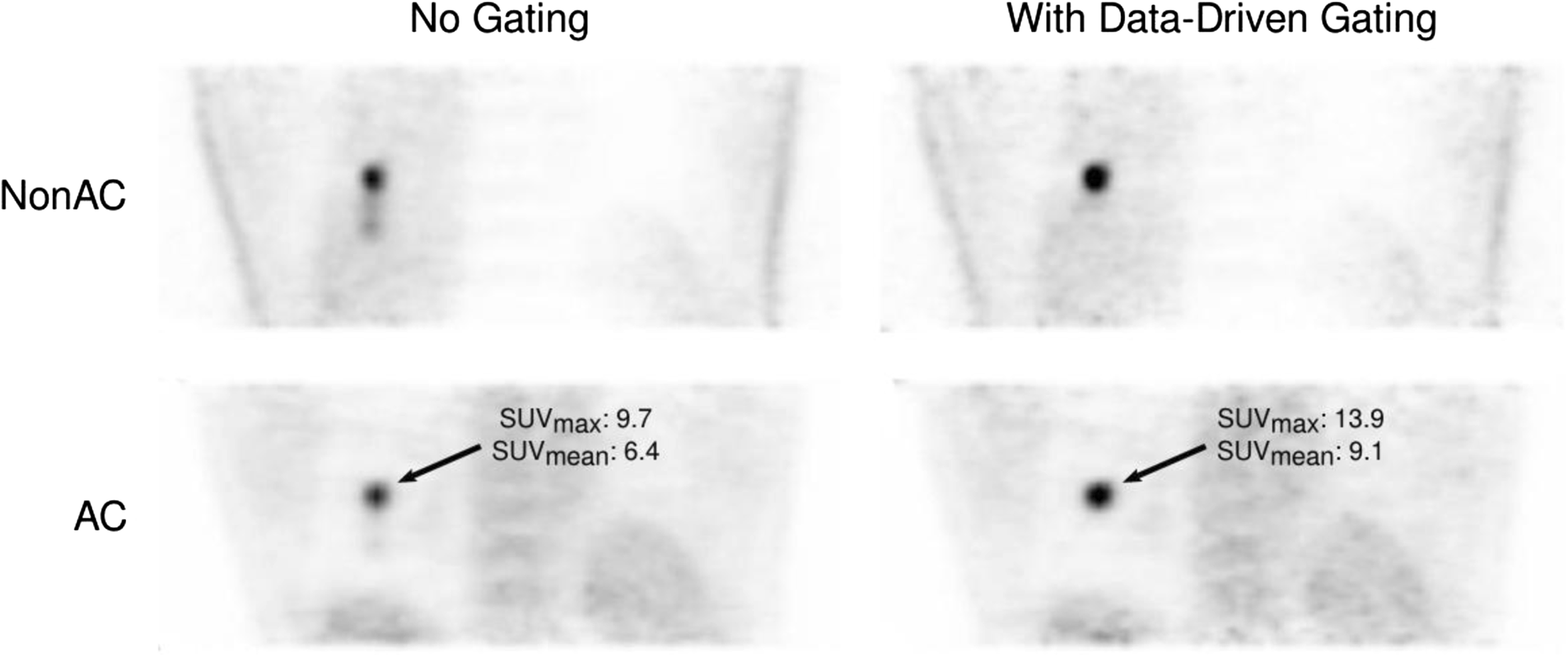

Supplement: Supplementary file 3 — Authors’ original file for figure 1 [file 40658_2014_7_MOESM3_ESM.tiff]

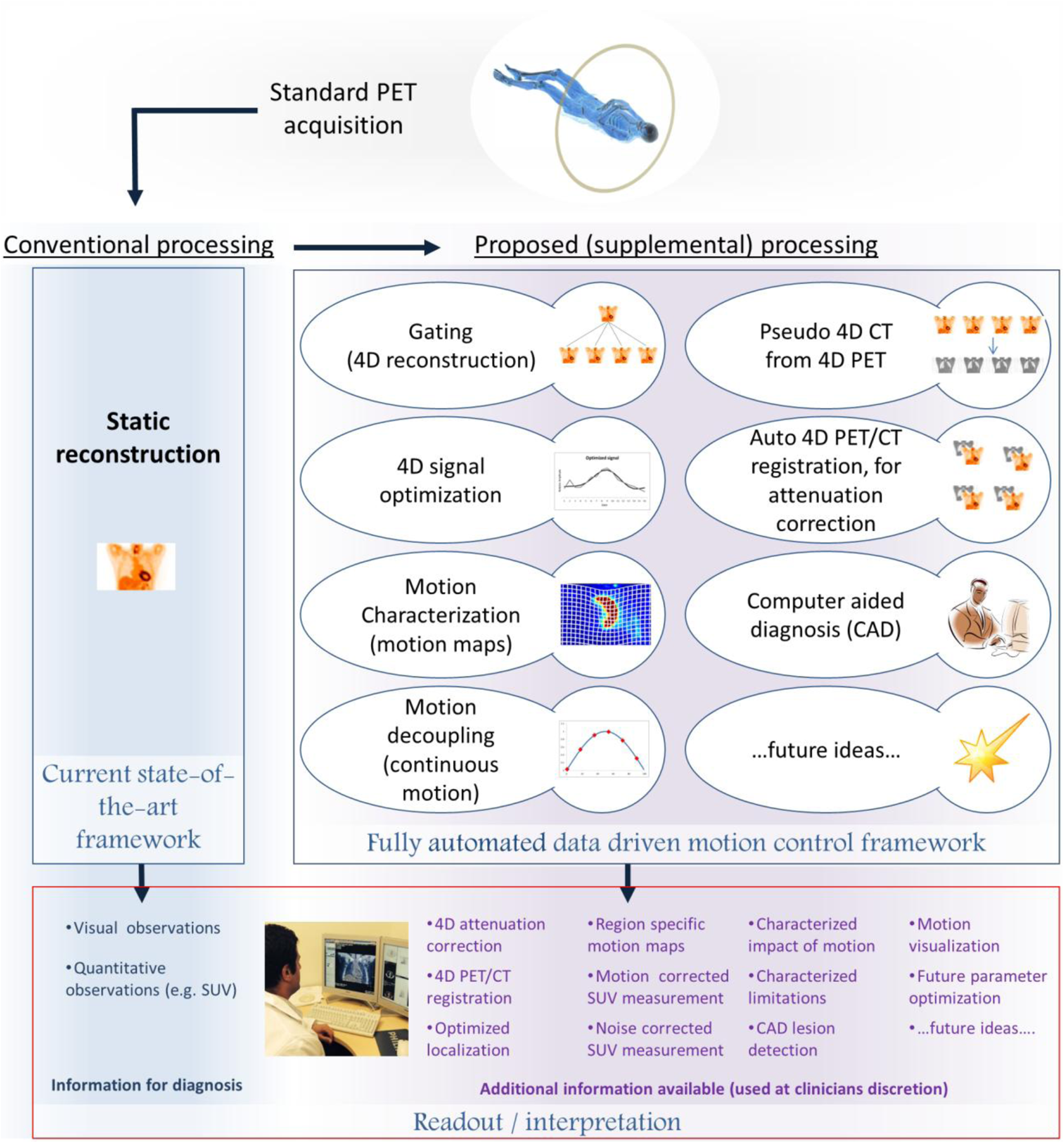

Supplement: Supplementary file 4 — Authors’ original file for figure 2 [file 40658_2014_7_MOESM4_ESM.tiff]

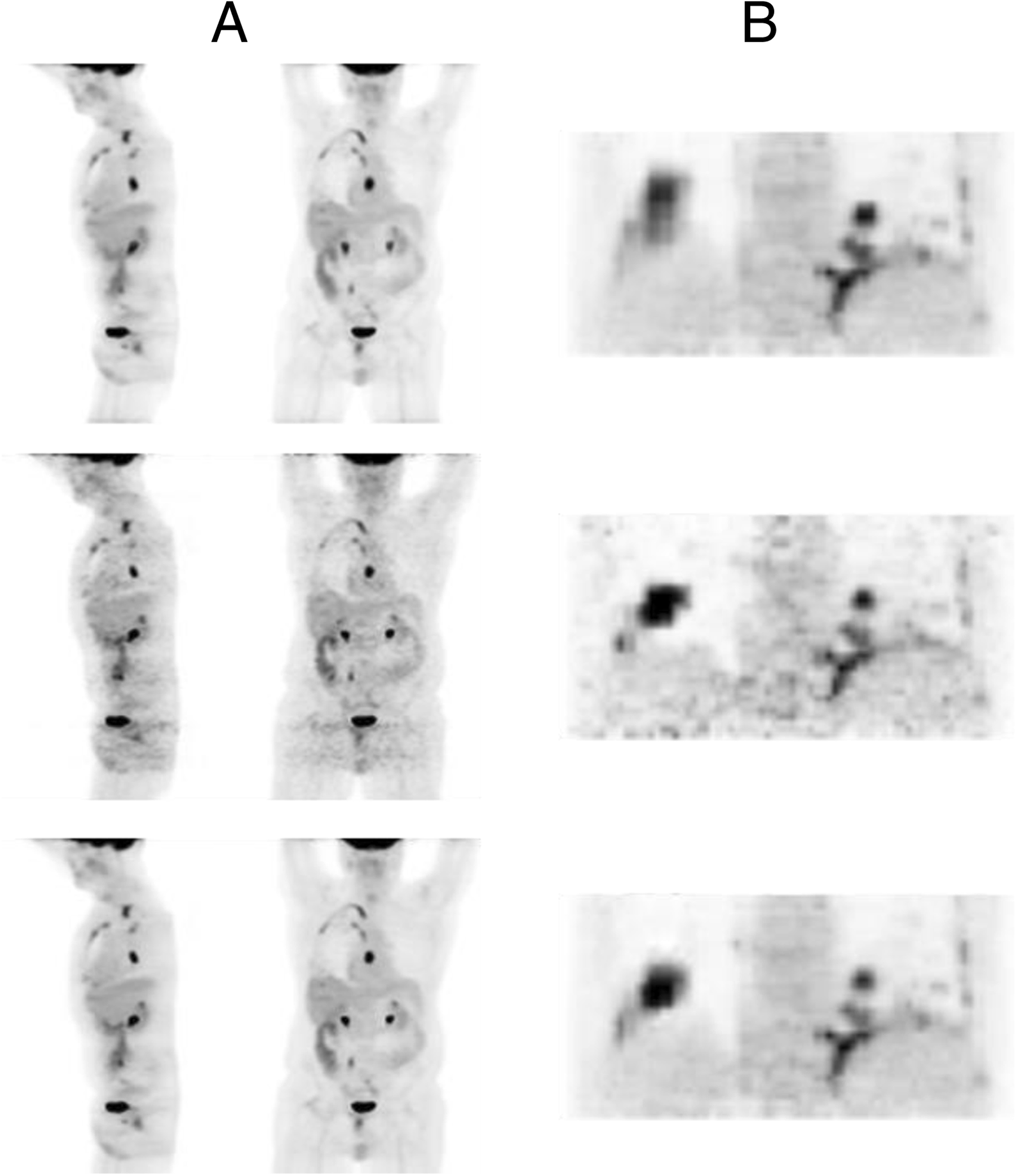

Supplement: Supplementary file 5 — Authors’ original file for figure 3 [file 40658_2014_7_MOESM5_ESM.tiff]
