# Supplementary material for: On transcending the impasse of respiratory motion correction applications in routine clinical imaging - a consideration of a fully automated data driven motion control framework
Source: EJNMMI Phys. 2014 Jun 17;1:8. doi: 10.1186/2197-7364-1-8 (PMC4673082; doi:10.1186/2197-7364-1-8)
Supplement: Supplementary file 6 — Authors’ original file for figure 4 [file 40658_2014_7_MOESM6_ESM.docx]

Online figures A and B are multiframe animations that illustrate the motion and noise properties present in the data sets mentioned in Figure 1. Animations are displayed at 30 frames per second to support the 90 frames/respiration period generated in the signal optimized images.

Both animations can be downloaded by pasting the following link into a browser address bar: <http://bit.ly/1gVDzEr>. They are .avi filetype.

| 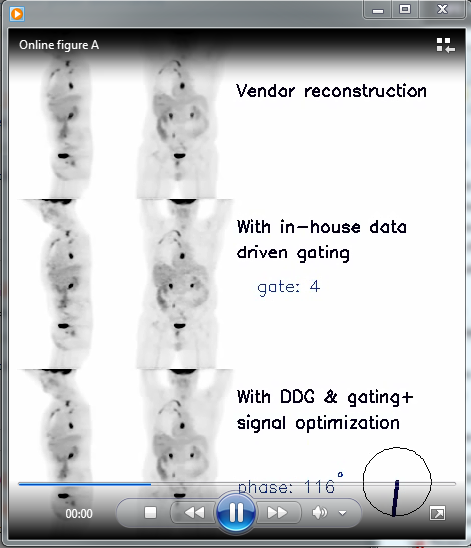 | 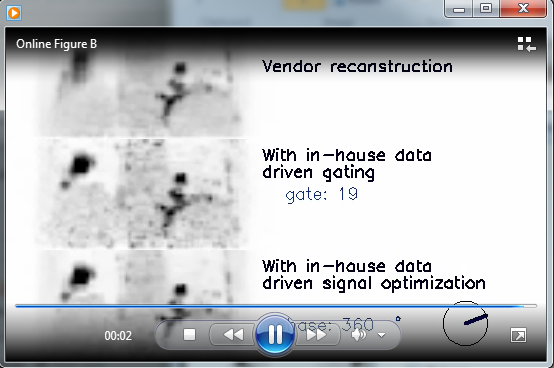 |
| --- | --- |
